# Recurrent Sigmoid Volvulus in Children—Our Experience and Systematic Review of the Current Literature

**DOI:** 10.3390/children10091441

**Published:** 2023-08-24

**Authors:** Jonathan Hencke, Steffan Loff

**Affiliations:** Department of Pediatric Surgery, Olgahospital, Klinikum Stuttgart, 70174 Stuttgart, Germany

**Keywords:** sigmoid volvulus, acute abdomen, Hirschsprung disease

## Abstract

Sigmoid volvulus (SV) occurs rarely in children. After encountering two cases of recurrent SV, we reviewed the literature to define the recurrence risk, identify outcome predictors and to give treatment recommendations. We found 256 cases reported in children (mean age 10.2 years, gender ratio (m:f) 2.3:1). Associations exist with Hirschsprung disease (HD) in 10%, neurodevelopmental disorders in 10.9% and chronic constipation in 10.2%. Common symptoms and clinical signs were abdominal pain (85%), distension (85%), tenderness (54%) and vomiting (59%). Signs of peritonitis were present in 14% and indicated a gangrenous sigmoid (*X*^2^ = 45.33; *p* < 0.001). A total of 183 had abdominal radiographs, and 65% showed a positive ‘coffee-bean-sign’. Contrast enemas were positive in 90%. A total of 124 patients underwent laparotomy; in 41 cases, the sigmoid was gangrenous and associated with more complications (*X*^2^ = 15.68; *p* < 0.001). Non-operative treatment (NOT) like endoscopic, fluoroscopic or rectal tube decompression was performed in 135 patients and successful in 79% with a 38–57% recurrence rate. A total of 73 patients subjected to elective surgery: 50 underwent sigmoid resection; 17 had surgery for HD. Clinicians should consider SV in all children with abdominal pain, distension and vomiting. Gangrene carries a higher morbidity. After successful NOT we recommend counselling about the recurrence risk and definitive surgery should be advised. HD is frequent in newborns but sometimes found in older children.

## 1. Introduction

Although a common cause of large bowel obstruction (LBO) in adults [1], sigmoid volvulus (SV) seems to rarely occur in children as previous reports show [2]. This acute condition involves an internal torsion of the intraperitoneal, mobile sigmoid colon around its mesentery, leading to LBO and impaired perfusion. It is facilitated by a long, redundant sigmoid, often occurring with known predisposing factors like chronic obstipation, diabetes, nutrition habits and Chagas disease. In children, reported pre-existing conditions are chronic constipation, neurological diseases including mental development delay and myopathies, chronic intestinal pseudo-obstruction (CIPO), Chagas disease and Hirschsprung disease (HD).

The typical clinical signs are abdominal pain and distension, failure to pass stool or gas or vomiting. The diagnostic work-up consists of plain abdominal radiographs and, in some cases, an additional contrast enema (CE) [3]. The common sign of an SV on an abdominal radiograph is the ‘coffee-bean sign’ resembling the distended, air-filled sigmoid which is obstructed on both its proximal and distal end [4]. Furthermore, radiographs may include signs of a perforation or ileus. Because abdominal computed tomography (CT) with or without contrast is a common diagnostic tool in acute bowel obstruction, this may also show pathological signs of SV like the ‘whirl-pool sign’ or the ‘bird’s beak’, also a common finding in CEs [3].

For the acute treatment of SV in adults, the established approach is to attempt to decompress the sigmoid either colonoscopically, with a sigmoidoscope or rectoscope or with a large bore rectal tube. In an unsuccessful attempt or if necrosis is suspected, open surgery would be conducted with manual detorsion and, if appropriate, a sigmoidopexy or sigmoid resection with a primary anastomosis (SRPA) or a colostomy depending on the condition of the bowel [5].

Because recurrences occur frequently in adults [6], the established definitive treatment aims to stabilize the sigmoid and prevent another torsion. The treatment may be either SRPA or sigmoidopexy, procedures which can be performed open or laparoscopically. Before the definitive treatment takes place in children, HD should be excluded through a rectal biopsy [7].

The diagnostic work-up and treatment of SV in children is often based on the experience in adults due to the lack of larger studies. With this systematic review, we aim to investigate the parameters of pediatric SV as the rarity of this condition hinders the implementation of controlled studies. The compiled data should bolster our diagnostic and therapeutic recommendations.

## 2. Materials and Methods

For this review, the inclusion criteria were publications available online reporting on new/own cases of SV not published yet elsewhere, patient age ≤ 18 years and diagnosis of SV confirmed radiologically and/or at surgery or autopsy. We excluded reports of mixed populations with adults which did not clearly identify the number of children with SV, cases of ileosigmoid knotting without confirmed SV and cases of colonic volvulus other than SV.

We searched the databases PubMed/MEDLINE, Scopus and Google Scholar with the keywords “sigmoid volvulus” and “children”, the last searches were in June (PubMed) and August 2023 (Scopus and Google Scholar). In Pubmed and Scopus, all search results were screened, while in Google Scholar, only a maximum of 500 search results per each 10 year interval from 1900 to 2023 were screened. Screening of the title, type of article, abstract and text excerpts (in Google Scholar) was conducted manually by the first author. Articles which met the inclusion and exclusion criteria were then selected. Articles which could not be retrieved for review were then excluded as well as cases which were clearly already reported on by the same author. Non-English publications were translated if they were accessible.

All included and retrieved publications were reviewed manually by the first author for the following measures: number of cases of SV; gender of patients; patient age; underlying conditions (especially HD, neurological or neurodevelopmental conditions or chronic constipation); leucocyte count; the presence of abdominal pain, vomiting, constipation or diarrhea as a reported symptom; the clinical finding of abdominal distension, tenderness, an abdominal mass or clinical peritonitis; if an abdominal radiograph was obtained and if it presented a ‘coffee-bean sign’; if a contrast enema or a computed tomography was conducted and if it was diagnostic of SV; if the SV was not suspected prior to surgery and was discovered intraoperatively; the use of colonoscopy, rectal tube, fluoroscopy or (rigid) sigmoidoscopy for SV detorsion and its success; the number and timing of an SV recurrence; the encountered condition of the sigmoid (viable vs. gangrene); the type of surgery conducted in either an emergency or elective setting; complications and outcome, e.g., fatalities.

Reports were only included in the analysis of a certain measure, if the measure was stated in the report, e.g., if the report did not specify on symptoms, it was excluded from this analysis and not counted as ‘absence of symptoms’. Exceptions are complications and recurrences which were often only reported if they occurred.

Analysis of the results was conducted by calculating the mean age and gender ratio, percentage of the associated conditions and plotting the correlation with the corresponding age, calculating the percentage of symptoms and clinical signs present, the percentage of diagnostic abdominal radiograph, CE and CT, and the success rates for non-operative treatment and its recurrence rates. Surgical methods are presented in tables and grouped with the intraoperative finding of a viable vs. a gangrenous sigmoid. The statistical significance of an association was determined using the Chi^2^-test and Fisher’s exact test for the following associations: age < 6 months with HD, gangrene with complications and mortality, symptoms, clinical findings and leucocyte count with the presence of gangrene; for these, also predictive values and Odds Ratios were generated.

As all publications were case reports or series, a risk of bias assessment was not conducted for the individual reports but for this review. The review was not registered beforehand and there was no explicit protocol prepared before.

Two own cases of sigmoid volvulus from our institution between 2019 and 2020 are presented. Written consent including the use of images was obtained from the parents.

## 3. Case Presentation

### 3.1. Patient 1

A four-year-old boy was presented to our interdisciplinary pediatric emergency department with unrest and supposed abdominal pain since the day before. His last bowel movement was relatively firm and the mother had to help digitally. On the day of admission, the patient vomited dark fluid three times in the morning.

The boy has a medical history with a neurological delay of unknown origin with muscular hypotonia and hypoplasia of the corpus callosum. He has had focal epileptic seizures in the past. Furthermore, the boy has a long history of constipation and takes four grams of Macrogol daily.

Upon examination the patient was alert and showed stable vital signs. The abdomen was enormously distended and tender but without guarding. Bowel sounds were almost absent.

**Figure 1:**
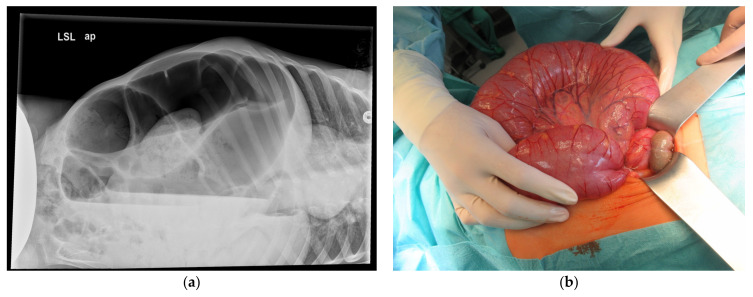
Patient 1, acute sigmoid volvulus: (**a**) lateral decubitus radiograph with ‘coffee-bean-sign’; (**b**) corresponding intraoperative finding.

Given the suspected bowel obstruction, a plain abdominal radiograph in lateral decubitus position was performed (Figure 1a), showing the classic ‘coffee-bean-sign’ with a sigmoid expanded to a diameter of 6.2 cm, reaching into the left upper quadrant, accompanied by coprostasis in other sections of the bowel.

Because a perforation could not be excluded, an open approach was decided. A sparing median laparotomy was performed, and the dilated bowel could be ventralized through the incision (Figure 1b). The elongated sigmoid showed a 360° SV and no signs of necrosis or perforation. After detorsion and manual antegrade decompression, the complete bowel was examined. A Meckel’s diverticulum with a considerably long and narrow configuration and therefore a possibly increased risk for inflammation was detected and stapled off.

The postoperative period was uneventful. Oral feeds began on the second postoperative day; stool was passed the day after. The boy was discharged home after five days in hospital. The parents were counselled about a possible recurrence and further work-up was recommended but did not take place.

About two and a half months after the first event, the patient presented again with recurring vomiting over the past four days and absent bowel movements for one day. Upon presentation the abdomen was similar to the first presentation—distended, tender, no guarding, almost no bowel sounds.

Because a recurrent SV was suspected, a plain abdominal radiograph was taken. Intraperitoneal air could be excluded, and the boy was prepared for an emergency colonoscopic decompression under general anesthesia. The torqued sigmoid loop could easily be reached with the colonoscope; the bowel wall was without any signs of ischemia, yet it seemed more perfused indicating venous congestion. The colonoscopy was conducted up to the proximal end of the loop. Biopsies were performed. The postoperative radiograph no longer showed signs of SV.

HD was suspected due to the severe chronic constipation and full-thickness rectal biopsies were performed but had to be repeated because they were not diagnostically conclusive with one specimen being lost and another one not being a sufficient sample. In the end, the biopsies excluded HD and only showed changes of the mucosal structure consistent with chronic constipation. A contrast enema demonstrated an enormous sigmoid and no typical signs of HD.

We opted for an elective sigmoidectomy to remove the redundant sigmoid and prevent a recurrence. We chose an open approach with a short median incision and a hand-sewn end-to-end anastomosis, removing 35 cm of dilated, redundant sigmoid. The postoperative period was uneventful except for an epileptic seizure requiring adjustment of the patient’s daily levetiracetam dose. There have been no recurring events of SV. The histology of the sigmoid showed hypoganglionosis of the colon characterized by small ganglia in the myenteric plexus with a maximum of 15 ganglion cells per ganglion (but most a lot less) and interganglia distances of more than the doubled ganglion diameter; no architectural changes in the muscular layer indicating an inflammatory or degenerative cause were noted.

### 3.2. Patient 2

**Figure 2:**
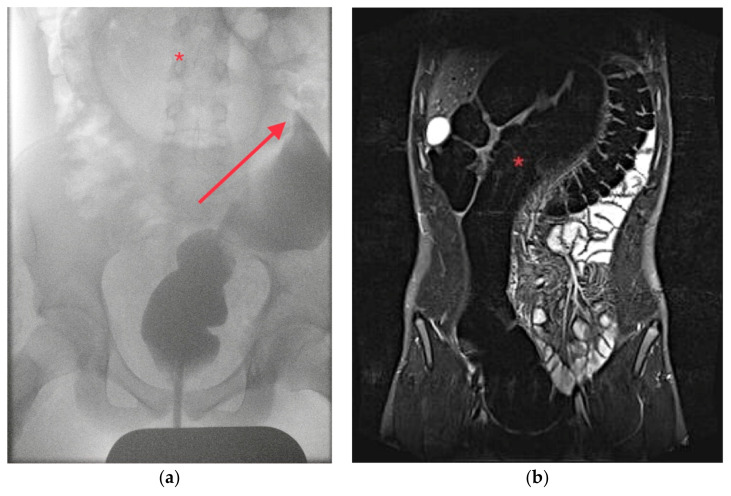
Patient 2: (**a**) Contrast enema showing the air-filled, dilated sigmoid (*) and the ‘bird’s beak-sign’ (arrow); (**b**) MRI showing a grossly enlarged and dilated sigmoid (*).

A 14-year-old adolescent boy presented to our interdisciplinary pediatric emergency department with severe colicky abdominal pain since the day before. The abdomen was distended and mildly tender without guarding. The medical history was without any hints for HD or chronic constipation. Ultrasound showed dilated, air-filled bowel loops, so an abdominal radiograph was performed suspecting LBO. The radiographic findings were pathognomonic for SV without signs of perforation. A decompression with a large bore rectal tube could be performed. Upon request by the parents, the patient was discharged home with recommendation for a follow-up visit a few days later, including a diagnostic work-up. However, the patient presented again the next day with similar abdominal pain and a radiograph confirmed the recurrent SV. Another decompression by rectal tube was attempted but was only successful after performing a contrast enema under fluoroscopy. (Figure 2a). Two days later, the patient presented one more time with the same symptoms, and again, decompression under fluoroscopy with a contrast enema was successful. The patient was admitted and underwent an MRI and a colonoscopy to rule out any rare anatomic conditions or neoplasms like polyps causing the uncommon finding of SV without any prior history of constipation. HD was considered highly unlikely so rectal biopsies were omitted. The MRI showed a considerably large and thick sigmoid (Figure 2b).

**Figure 3:**
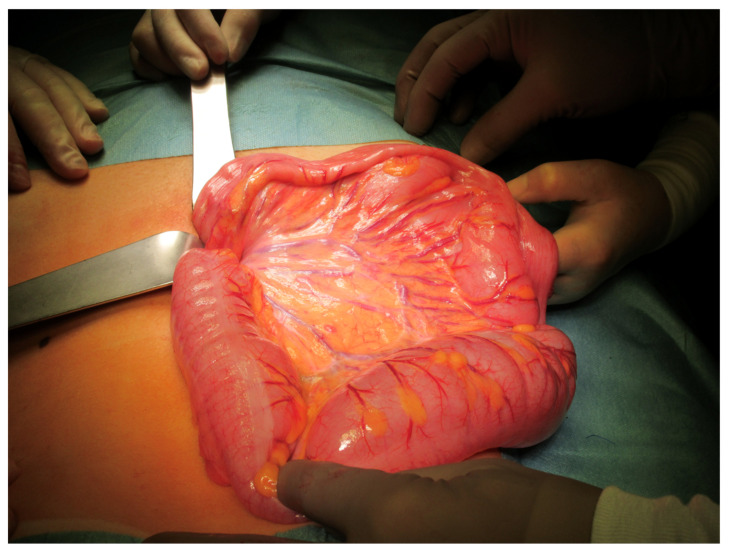
Patient 2: redundant sigmoid (45 cm) during elective sigmoidectomy.

The patient underwent an early elective sigmoidectomy within the same hospital stay, using a short laparotomy with laparoscopic assistance. An amount of 45 cm of redundant sigmoid was removed (Figure 3) and a hand-sewn end-to-end anastomosis was performed. The postoperative period was uneventful. The specimen of the removed sigmoid showed dilated, hypoganglionotic bowel without architectural changes of the muscularis or mucosa. Only small ganglia in the myenteric plexus with an interganglia distance of more than twice the ganglion diameter were present. The origin of the hypoganglionosis remains unclear, and a pathogenesis secondary to an undetected necrotizing enterocolitis or chronic dilatation was discussed by the pathologist. Upon follow-up, the patient had regular bowel movements and no recurrence of SV.

## 4. Results of Literature Review

### 4.1. History

Sigmoid volvulus has been known in the adult population for centuries, but the oldest article available online reporting on a pediatric case was written by Alexius McGlannan in 1915 [8]. In 1931, Carnes Weeks published the case of an 18-year-old boy and also provided a list of other previous cases of SV, including nine children [9]. Another report by Allen, Nordstrom et al. in 1964 already mentioned a non-operative emergency treatment with fluoroscopy [10]. Rectal tube decompression and sigmoidoscopy use in children were published in 1974 by Wilk, Ross et al. [11] and were a regular treatment from that point on. Colonoscopic detorsion in an adult patient was first published by Ghazi, Shinya et al. [12], but the first reported use in children only took place in 2004 [13]. Although open detorsion was the common approach in the first reports [8,14,15], elective surgery to prevent a recurrence was already reported in 1964 [16] but became more regularly used in the 1990s.

### 4.2. Selection Results

**Figure 4:**
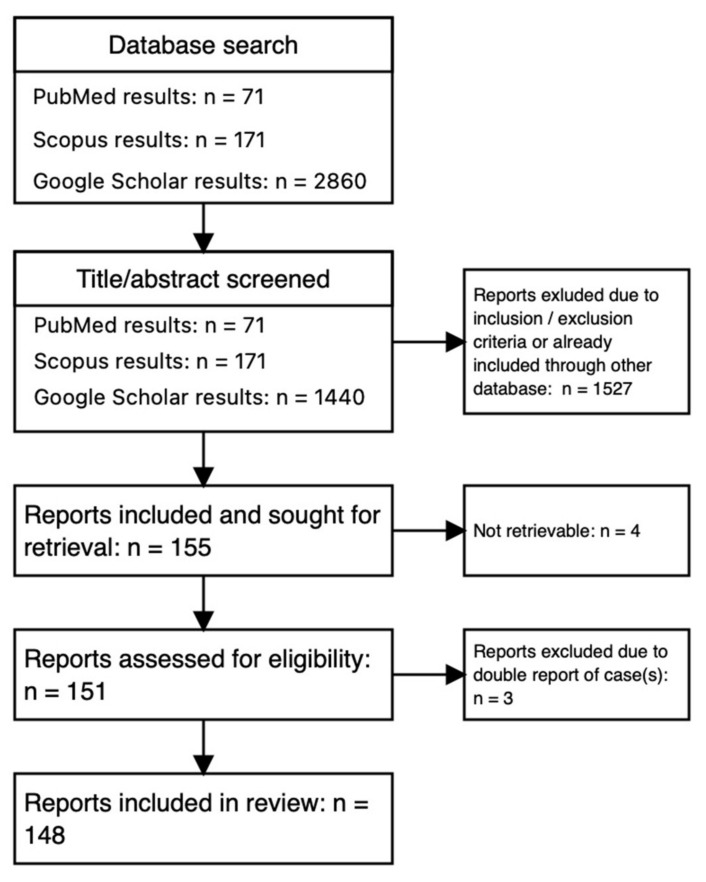
Flowchart of the systematic review.

The results of the database search, screening and selection process are outlined in Figure 4. Three reports were excluded in the last step as they were clearly identified as cases already presented by the same author(s). The type of articles selected included case reports, case series without controls, clinical images and letters to the editor. Two case reports included a comprehensive review of the existing cases up to the respective year (1990 and 2000).

Most publications until now are case reports with one or two cases and so far, there are only few series; most notable by Atamanalp et al. (19 patients) [17]; Colinet et al. (13 patients) [18]; Destro et al. [19] (8 patients); and Puneet et al., Chirdan et al. and Khalayleh et al. (6 patients each) [4,20,21].

Many reports did not include all measures investigated in this review, and especially in larger case series of populations mixed with adults, the results were incomplete. Only 51 publications with a total of 59 patients provided details for all measures.

### 4.3. Epidemiology

**Figure 5:**
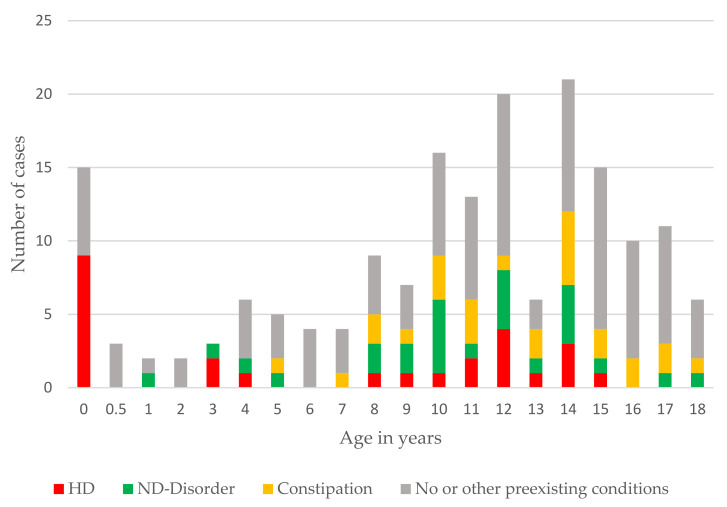
Distribution of age and association with other medical conditions.

We found 148 publications reporting a total of 256 cases of SV in children aged 1 day up to and including 18 years. The mean age was 10.2 years and SV occurred in 144 males and 63 females, respectively, making the gender ratio (m:f) 2.3:1. A wide range of preexisting medical conditions was reported (Figure 5) and differed according to the age of the patients. HD was reported in 27 patients (10%) and is significantly more likely to be diagnosed in infants and neonates (9/15 cases under 6 months; OR = 14.29, *X*^2^(1, *n* = 194) = 30.42; *p* < 0.001), but also appears in older children and adolescents. Mental disability, neurological or developmental disorders were encountered in 28 patients (10.9%). Chronic constipation without other known conditions was preexisting in 26 patients (10.2%).

Other, more rarely encountered predispositions included Chagas disease (one case) [22], anal stenosis (one case) [23], imperforate anus and sigmoid duplication (one case) [24], prune belly syndrome (one case) [25], chronic intestinal pseudo-obstruction (CIPO/two cases) [18,26] or atypical interenteric adhesions (one case) [27]. However, the majority (147 patients, 57.4%) had no known conditions reported.

### 4.4. Symptoms and Clinical Presentation

The symptoms and clinical presentations of childhood SV have been studied before in reviews and have been reported in large series (Table 1). In the previous articles, the most prominent symptoms were abdominal pain in 66–100% of patients and vomiting in 31–74%. Obstipation (10–58%) and diarrhea (8–23%) were reported less frequently. Abdominal distension was encountered in 56–84% and tenderness in 17–100%. An abdominal mass was palpated in 3–10% of patients. The presence of peritonitis differed considerably between 53% and no peritoneal signs at all. Our review is generally consistent with these findings, showing abdominal pain in 85%, vomiting in 59%, constipation in 56% and diarrhea in 10% of cases. Clinical findings were abdominal distension in 85%, tenderness in 54%, abdominal mass in 3% and peritonitis in 14%.

Clinical signs of peritonitis increase the likelihood of a gangrenous sigmoid significantly (Table 2). Abdominal tenderness is also more associated with gangrene; however, it is not as significant as clinical peritonitis. An elevated leucocyte count is an indicator for gangrene; specifically, no patients with gangrene had a normal leucocyte count.

### 4.5. Imaging

Plain abdominal radiographs were obtained in 183 patients; in 119 cases, a positive ‘coffee-bean-sign’, ‘omega-sign’ or a typical U-shaped dilated sigmoid was found, suggesting SV in 65% of radiographs. Contrast enemas took place in 69 patients with positive findings for SV in 62 patients (90%). A total of 39 patients underwent a computed tomography (CT) which was always diagnostic, most often with a ‘whirl-pool-sign’ of the mesosigmoid. A total of 27 patients had a laparotomy for suspected diagnoses other than SV; 21 for not specified bowel obstruction, 2 for intussusception, 1 for a suspected Burkitt’s lymphoma and 3 for appendicitis.

### 4.6. Treatment

Initial non-operative treatment was reported for 135 patients (rectal tube: 22, fluoroscopy: 30, (rigid) sigmoidoscopy: 24, colonoscopy: 59) with an overall success rate of 79 % (Table 3). Spontaneous reduction was observed in one case [28]. Recurrences after successful non-operative treatment were reported in 41 cases (38%); however, 35 patients underwent early definitive surgery after their first episode of SV. If we exclude those treated before any recurrence could occur, the recurrence rate rises to 57%. The recurrence occurred within a broad timeframe: in eight cases, the SV recurred within a week, in twelve patients between one and six months, while in seven cases the time to recurrence was longer; in the longest case, recurrence occurred four years after the first event.

Some patients had simultaneous findings like ileosigmoid knotting (11 cases) [17,20], splenic torsion (2 cases) [19,29] or ileocolic intussusception (1 case) [30]. A volvulus occurred in other intestinal sites at an earlier or later stage in three cases: one with volvulus of the transverse colon two years earlier [31], one with cecal volvulus later [32] and one with both cecal and transverse colonic volvulus later [18].

A total of 124 patients had emergency operative treatment; in 41 patients, gangrene of the sigmoid was encountered, whilst 83 patients had a viable sigmoid (Table 4).

Of the patients with a viable sigmoid, 20 patients underwent open detorsion alone, in 8 patients an additional sigmoidopexy took place, 2 received an extraperitonealisation of the sigmoid and 2 a mesosigmoidoplasty to widen the mesosigmoid. In 41 patients, the redundant sigmoid was resected: with a primary anastomosis in 35 cases and colostomy formation in 6 cases. Three patients with suspected HD received open detorsion with formation of an ileostomy.

A total of 25 patients with gangrene were treated with a Hartmann’s procedure, 10 with a resection and another type of colostomy and 6 received a primary anastomosis after the resection.

A total of 73 patients underwent elective surgery after SV (Table 5); in 26 cases, this took place after a recurrence. The procedures were SRPA in 49 patients, and in 1 patient this was accomplished transanally [33]. Four patients received a resection with colostomy formation (one due to CIPO) and one a total colectomy [18,19]. A total of 17 patients underwent definitive surgery for HD (Swenson procedure: 2; Duhamel: 2; Yancey–Soave: 5; other or not specified pull-through: 8)

### 4.7. Complications and Outcomes

Re-operations for a recurrence after emergency resection or fixation were never necessary. However, one patient required revision for rectal necrosis after colostomy formation [34], and another one for a leaking dehiscence after primary anastomosis [22]. Adhesive bowel obstruction was reported in two patients after either open detorsion or after resection of a gangrenous sigmoid with primary anastomosis [28]. An enterocolic fistula was noted in one case [35]. Short bowel syndrome as a consequence of compression of the mesentery by the SV and therefore enteral ischemia was a complication in one case [36]. Persisting or a new onset of constipation was reported in two patients. Elective surgery overall had almost no complications; the only reported complication was an anastomosis stenosis after total colectomy [19] and two cases of anastomosis dehiscence after SRPA. Gangrene increased the rate of complications significantly (11/41 vs. 13/200; OR = 5.27; *X*^2^(1, *n* = 241) = 15.68; *p* < 0.001). If assessing the rate of overall complications after definitive treatment (sigmoidectomy w/ primary anastomosis or sigmoidopexy) either in an emergency or an elective setting, we find only slightly more complications in an emergency, also not at a significant level (5/53 vs. 3/51; OR = 1.6; Fishers exact test, *p* = 0.716).

Fatal outcomes after surgery were reported in nine cases; three from shock in the presence of gangrene, one from peritonitis after a leak after primary anastomosis [17] and one from wound dehiscence [20]. Four patients died of shock after surgery although a viable sigmoid was found and detorsion took place. Statistically, cases with gangrenous sigmoid were also significantly more likely to have a fatal outcome (4/41 vs. 6/200; OR = 3.5; *X*^2^(1, *n* = 241) = 3.90; *p* = 0.049). Furthermore, there are three fatal cases without treatment in the literature [37,38,39], emphasizing that SV is a potential lethal condition if left untreated.

### 4.8. Risk of Bias Assessment

Selection bias: As the selection process aimed to include a maximal number of cases, the inclusion and exclusion criteria were relatively broad. The number of cases considered here surpasses previous reviews clearly. Because all articles were reviewed manually, it is unlikely that cases were included which should not have been. In contrast to this, there is a considerable possibility that reports were omitted in the screening process due to unclear phrasing in the title or abstract. Reports missed because of missing online availability or limited indexing in databases can also be presumed. Therefore, the risk of selection bias is determined to be at least moderate.

Reporting bias: Of the literature included, there were 120 case reports including letters to the editor, clinical image, 20 case series and 8 series of a different topic, which included cases of SV, etc. Especially these case reports have a high risk of reporting bias as only remarkable cases with interesting outcomes become published. This may be slightly reduced in case series or when a large series of, e.g., all cases of LBO is published with several cases of SV, as this should have a lesser influence on the publication of an individual case. Nevertheless, the risk of reporting bias and in particular the effect of publication bias has to be considered high in this review.

Attrition bias: As outlined in 4.2, the majority of reports lacked all parameters for a full investigation. Excluding these cases from the review would have increased the selection bias considerably and reduced the data supporting this review’s results. The missing measures decrease the sample size for the relevant parameters and may lead to biased results not representing the population. The measures most often excluded in the publications were laboratory values (missing in 196 patients), symptoms (missing in 45 patients) and clinical findings (missing in 49 patients). The duration of follow-up was seldom indicated and varied between three months and several years; some complications and recurrences may be consequently missed. As a result, the risk of attrition bias is presumed high.

Performance bias: As there were no controlled studies included (and available on this topic), this is not applicable for this review.

### 4.9. Level of Evidence

As the available literature for this topic consists exclusively of case reports and case series without cohort or case-control studies, the level of evidence of both the literature and this review is low (level V).

### 4.10. Methodological Quality Assessment of the Literature

The selected reports were assessed with the JBI tool and showed generally valid results. The first three points were clear in all reports where applicable. For inclusion of participants (point four and five), only case series were assessed, with favorable results in eight series, unclear inclusion in two and no complete and consecutive inclusion in one series. Demographics of the patients were unclearly reported in eight reports and not included in five, but they were clearly stated in the remaining publications. The clinical information of the individual patients was slightly incomplete in nine articles and only sparse in ten. Follow-up was incomplete or unclear in 27 reports and outcome information was completely missing in 26 articles. In 46 publications the presenting site’s or clinic’s location or demographic was not stated in the text. Statistical analysis was limited to case series and appropriate in five articles and not conducted in the remaining ones.

## 5. Discussion

SV is the most common type of colonic volvulus (CV) in both children and adults [5,19]. It is facilitated by a long, redundant sigmoid with a narrow base [1]. SV occurs in children of all ages, from newborns to school-age children and adolescents. There seems to be a two-peak distribution as shown in Figure 4: the first peak in the first six months of life and the second peak in school-aged children. We found a male predominance at 2.3:1, which is slightly lower than the review by Smith et al. from 1990 [25] and the 3.5:1 from a review from 2000 [2].

In newborns and infants, we found a strong association with HD as almost 2/3 of reported cases under one year had positive rectal biopsies for HD. Interestingly, a small number of older children aged 9–15 years presented with SV as their first complication or manifestation of HD. The obstructive component of HD with dilatation of the sigmoid seems to play a role in the pathogenesis of SV in HD unlike in other conditions, as Destro et al. showed a difference in colonic diameter in patients with CV with and without HD [19]. The incidence of HD in pediatric SV in our review is 10%, the previous review by Salas et al. from 2000 has noted an incidence under 18 years of 17% [2], while the percentage of patients presenting with SV in the HD population is significantly lower with 0.6–3% [21,40]. Most recently, Uylas et al. published a review on HD and SV in patients of all ages, showing a number of adults until the age of 82, but mostly under 40, with SV as their first presentation of HD [41]. Some of the larger existing series on pediatric SV, however, included not a single case of HD [17,18,20]. As first recommended by Sarioglu et al. and Venugopal et al. in 1997, exclusion of HD by rectal biopsy should take place before definitive treatment [7,40]. HD was considered in both our patients and we took effort in excluding it especially in the first patient due to a long history of constipation. The rectal biopsy in the second patient was omitted because he had no history of constipation and to prevent a delay in the definitive treatment after several recurrences of SV within a short period.

Another known association of SV exists with neurological and neurodevelopment disorders (NDs) [31]. We found NDs to be present in 10.9% of the reviewed cases, with a range of different NDs including autism spectrum disorder, myopathy, cerebral palsy or non-specified mental development delay or disability. Causality of this predisposing factor varies: some authors suggest that constipation caused by nutrition and medication is a common feature of NDs and therefore leads to an elongated and dilated sigmoid [31], whilst others suspect the gut–brain axis (GBA) to play a role [19]. A stated argument for the GBA hypothesis is the reduction in enteric ganglion cells preceding a CV as shown by Fujiya et al. [42]. Consistent with this, hypoganglionosis was also histologically found in the removed sigmoid of both our patients.

Another aspect of the association of SV with ND concerns the possibly delayed diagnosis of SV due to the impaired ability of patients with ND to express their complaints and the often preexisting chronic constipation. Timely suspicion and prompt radiographic imaging can prevent a delayed diagnosis and adverse outcomes [43]. Our first patient can clearly be categorized as part of the patient population with NDs.

Chronic constipation (CC) without other causes was seen in 10.2% of the reviewed cases and has been described as both a cause of SV but also a symptom of the anatomy often present in SV patients. As the exact etiopathogenesis still remains unknown [19], both are possible: chronic constipation and/or dietary habits lead to an elongated sigmoid with narrow mesentery base or the chronic constipation is due to the elongated sigmoid. The latter possibly is the more suitable explanation as there have been a number of patients with these typical anatomic findings without a history of CC.

The symptoms and clinical presentation in children have been the subject of several reviews before. Patients most often complain of abdominal pain in more than three-quarters of cases and vomiting in more than half of cases. Constipation, obviously, has been reported as a symptom but also diarrhea in up to 10%. Usual clinical findings on examination were most often abdominal distension followed by tenderness. A more specific sign, an abdominal mass, was hardly ever noticed. Peritonitis is a clinical finding indicating complicated SV: they were present in 53% of patients in the series by Atamanalp et al. [17], in which 79% had gangrene of the sigmoid, but not in any patients in the series by Colinet et al. [18], where all 13 patients underwent endoscopic treatment. No clinical peritonitis is a good indicator for the absence of gangrene. In conclusion, there are no pathognomonic symptoms or clinical signs pointing towards the diagnosis of SV in children. Abdominal pain, vomiting, abdominal distension and tenderness can arise from a variety of pediatric abdominal conditions, with many having a higher probability than the unusual SV, i.e., intussusception, severe coprostasis, (perforated) appendicitis, midgut volvulus or adhesive bowel obstruction. A ‘textbook’ presentation with an acute onset of abdominal pain, distension and a palpable mass in the upper abdomen (the distended sigmoid), as it has been described in adults as von Wahl’s triad, can hardly ever be encountered in children. Vigilance for the possibility of SV matters and children with signs of bowel obstruction and those with suspected coprostasis failing to improve after enemas should undergo imaging, because the diagnosis of SV is most often obtained radiologically. The common imaging methods used are abdominal radiographs, contrast enemas (CEs) and computed tomography (CT) or magnetic resonance imaging (MRI). The classical sign, the ‘coffee-bean-sign’ was noted in 65% of the abdominal radiographs in our review; a previous review by Smith et al. found an even lower rate at 29% [25]. If a CE is obtained, it is diagnostic in 90% of cases. This is more accurate than the previously stated 61% in a mentioned review [25]. The cause of this improvement in sensitivity is unclear; radiologic appliances providing a higher image quality may be possible but also increased awareness of the possibility of SV. CEs bring another useful feature: after a positive finding of SV, an attempt to decompress and untwist the sigmoid can be undertaken immediately. CT is certainly a valuable tool and almost always diagnostic, but its use is more restricted in the pediatric population compared to its regular use in adults due to the radiation involved. An assessment for signs of complications like free abdominal fluid or gas is also possible in CT aiding in decisions for the adequate treatment.

Treatment is dependent on the presence or absence of complications, especially gangrene or an occurred or impending perforation. Since the publication by Bruusgaard in 1947, non-operative treatment (NOT) to accomplish emergency detorsion has been established [44] in adults and later accordingly in children with high success rates of about 80%. Colonoscopy is the standard treatment in adults [5]; however, this is not entirely supported in children, and a rectal tube or fluoroscopy/CE have similar success rates. Another aspect may be the availability: even in a relatively large pediatric center like our hospital, most colonoscopies are elective procedures and are hardly ever performed as an emergency treatment. Fluoroscopy or rectal tubes, however, are usually available even in small hospitals. NOT should therefore be attempted with the available means. If unsuccessful, resorting to a different procedure, i.e., switching from unsuccessful rectal tube decompression to fluoroscopy or endoscopy, is an option as this has been already reported in some patients and was also effective in our second patient.

Emergency surgery should be reserved for cases with suspected gangrene, perforation or those who do not respond to NOT attempts. An open approach is common with a detorsion of the sigmoid. There was only one reported recurrence after detorsion without any fixation in children. Our first patient is the second case raising the recurrence rate to 9.5%. However, the recurrence rate after open detorsion alone has been reported as 18.2% in the adult population [6]. Therefore, sigmoidopexy or resection even in the emergency setting is recommended in adults [5].

In the presence of gangrene, which is less often encountered (41/256 cases, 16%), a resection of the implicated sigmoid is required. A primary anastomosis—to spare the patient the burden of a colostomy and a second surgery—has been attempted in a small number (*n* = 6) of patients. Previous authors advised against it due to the possible complications of anastomosis dehiscence. Most cases of gangrenous sigmoid underwent a Hartmann’s procedure, certainly the safest option especially in the presence of shock due to prolonged and necrotic SV and possibly accompanying peritonitis. The reported deaths can be seen as lethal outcomes despite Hartmann’s procedure rather than being caused by it.

While some patients remain asymptomatic and without any recurrence after NOT, the overall recurrence rate can be considered high. The definitive treatment to prevent any recurrence is sigmoidopexy, SRPA or correction of HD. Smith et al. recommended routine SRPA for pediatric patients [25], while others concluded it should be reserved for recurrent SV. All methods of fixation of the mobile sigmoid like sigmoidopexy or extraperitonealisation had no reported recurrence in children, but there have been cases of recurrent SV after sigmoidopexy in adults [6], favoring resection over fixation. Nowadays, elective sigmoid resection often can be accomplished in a minimally invasive way, although a grossly enlarged sigmoid may hinder the feasibility and an existing CC can make extensive mechanical bowel preparation necessary. Our surgical method, which involves laparoscopic identification of the sigmoid and its eventration through a short periumbilical, median laparotomy followed by resection and a hand-sewn anastomosis outside the abdominal cavity, can be conducted without a stapling device and advanced laparoscopic skills and while still ensuring a safe anastomosis and minimizing contamination. The prolapsing, transanal technique described by Hamada et al. [33] is another useful method for SRPA especially for pediatric surgeons used to transanal pull-through for HD.

Because the time of recurrence of SV can be either early or late as shown in our two cases, we recommend counselling parents at the first occasion of SV after successful NOT about the risk of recurrence and offering an early elective SRPA. HD should be excluded through a rectal biopsy as early as possible, as this has been advised before [7,40]. Rectal biopsy can be omitted in patients who have a low risk for HD and for those where this would cause a delay until definitive treatment; an anorectal manometry with a positive trigger of the recto-anal inhibitory reflex (RAIR) may be sufficient and less invasive and time consuming.

**Figure 6:**
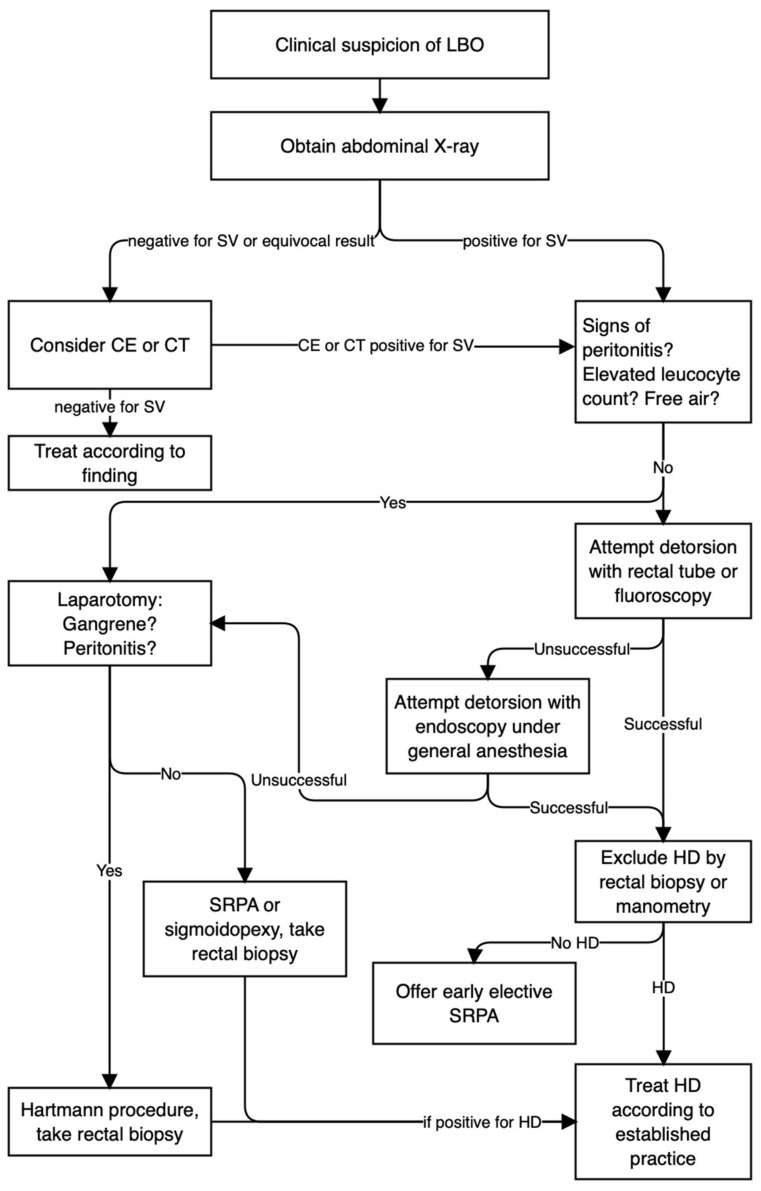
Proposed diagnostic and treatment algorithm.

An algorithm incorporating these suggestions for diagnostic work-up and treatment is outlined in Figure 6.

We are aware that there is possible improvement in the management of our presented cases. Therefore, one aim of this article is to outline the challenges and pitfalls, especially not to belittle the recurrence risk.

There are certainly significant limitations in this review: the actual number of childhood SV cases is probably considerably higher as many cases stay unreported. Some procedures have been used only a few times in children, so accurate descriptions of their safety or feasibility cannot be given. Several reported cases and complications happened many decades ago and in developing countries; anesthesia and perioperative care have improved immensely since then. Due to the rarity of SV in the pediatric population, there are no controlled or even randomized studies like in adults and these will not be available soon. Although a good number of reports and series already exist, continued presentation and retrospective analysis will help to understand this rare condition, establish standards of treatment and improve outcomes.

## 6. Conclusions

Clinicians should be aware of a possible SV in children of all ages presenting with abdominal pain, distension and vomiting, especially in school-aged boys and those with neurodevelopmental disorders or a history of constipation. Imaging, especially CT or CE, is usually diagnostic and NOT should be attempted with the available means if no contraindications are present. Delayed diagnosis and treatment with the development of gangrene carries a higher risk of morbidity and mortality. Normal leucocyte count and absence of clinical signs of peritonitis predict the absence of gangrene. After successful NOT, parents should be counselled about the high risk of recurrence and an early elective SRPA should be advised. The possibility of HD is higher in newborns and infants but should also be taken seriously in older children, as SV sometimes is the first apparent manifestation of HD.

## Figures and Tables

**Table 1:** Results of previous reviews, larger series and of our review: (**a**) reported symptoms; (**b**) clinical findings.

(a)	Abdominal Pain	Vomiting	Constipation	Diarrhea
Smith et al. [25] (1990 review)	26/39 (66%)	12/39 (31%)	4/39 (10%)	/
Salas et al. [2] (2000 review)	42/63 (67%)	30/63 (48%)	/	5/63 (8%)
Atamanalp et al. [17] (series)	17/19 (90%)	14/19 (74%)	11/19 (58%)	/
Colinet et al. [18] (series)	13/13 (100%)	7/13 (53%)	3/13 (23%)	3/13 (23%)
*Our review*	*180/211 (85%)*	*123/208 (59%)*	*113/202 (56%)*	*19/194 (10%)*
**(b)**	**Abdominal** **Distension**	**Abdominal** **Tenderness**	**Abdominal Mass**	**Peritonitis**
Smith et al. [25] (1990 review)	20/29 (69%)	12/29 (41%)	3/29 (10%)	2/29 (7%)
Salas et al. [2] (2000 review)	35/63 (56%)	11/63 (17%)	2/63 (3%)	/
Atamanalp et al. [17] (series)	15/19 (79%)	17/19 (90%)	/	10/19 (53%)
Colinet et al. [18] (series)	11/13 (84%)	13/13 (100%)	1/13 (8%)	0/13 (0%)
*Our review*	*175/207 (85%)*	*101/188 (54%)*	*6/195 (3%)*	*27/196 (14%)*

**Table 2:** Predictors for gangrene.

Predictor for Gangrene	Ratio	PPV	NPV	Sensitivity	Odd’s Ratio	Statistic Significance?
Clinical signs of peritonitis	11/19 vs. 8/146	58%	95%	58%	23.7	*X*^2^ (1, *n* = 165) = 45.33; *p* < 0.001
Abdominal tenderness	30/37 vs. 75/134	29%	89%	81%	3.37	*X*^2^ (1, *n* = 171) = 7.71; *p* = 0.005
Elevated leucocyte count	12/12 vs. 10/48	55%	100%	100%	∞	Fisher’s exact test, *p* < 0.001

**Table 3:** Non-operative treatment and its outcomes.

	*n*=	Successful	Recurrence	Definitive Surgery after Recurrence	Early Definitive Surgery	Recurrence Rate with Early Definitive Surgery Excluded
**Endoscopy**	83	65 (78%)	30 (46%)	19	20	66%
**Fluoroscopy**	30	24 (80%)	4 (17%)	3	7	24%
**Rectal tube**	22	18 (82%)	7 (39%)	4	8	70%
***Total***	*135*	*107 (79%)*	*41 (38%)*	*26*	*35*	*57%*

**Table 4:** Emergency surgical procedures for viable and gangrenous sigmoid and their outcome.

Condition of Sigmoid	Emergency Surgery	*n*=	Complications
a. Viable	Open detorsion	20	death: 3; constipation: 1; adhesive BO: 1
	Open detorsion w/fixation	8	
	Open detorsion w/ostomy	9	death: 1
	Resection w/colostomy	6	enterocolic fistula: 1
	Mesosigmoidoplasty	2	
	Resection w/primary anastomosis	35	death: 2; anastomosis dehiscence: 1
	Extraperitonealisation	2	
	Yancey–Soave	1	
	*total*	*83*	
b. Gangrene	Hartmann’s procedure	25	death: 3; SSI: 1; bowel necrosis: 1
	Resection w/colostomy	10	rectal necrosis: 1; SSI: 2; death: 1
	Resection w/primary anastomosis	6	adhesive BO: 1; SSI: 1
	*total*	*41*	

**Table 5:** Elective procedures after SV and their outcome.

Type of Procedure	*n*=	Complications
Resection w/primary anastomosis	49	anastomosis dehiscence: 2; constipation: 1
Swenson	2	
Yancey–Soave	5	
Duhamel	2	
Other or not specified pull-through for HD	8	
Transanal sigmoidectomy	1	
Correction of malrotation	1	
Resection w/colostomy	4	
Total colectomy	1	anastomosis stenosis: 1
*total*	*73*	

## Data Availability

Not applicable.
